# Study of Functional Outcome of Intra-articular Steroid Injection and Manipulation of Shoulder Joints in Frozen Shoulder

**DOI:** 10.7759/cureus.66727

**Published:** 2024-08-12

**Authors:** Himanshu Shah, Sujan Acharya, Anil P Yadav, Ashish Khadgi, Anupama Sharma

**Affiliations:** 1 Department of Orthopaedics, Buddha International Hospital, Ghorahi, NPL; 2 Department of Orthopaedics, Hetauda Hospital, Hetauda, NPL; 3 Department of Otolaryngology - Head and Neck Surgery, Lamahi Eye Hospital, Lamahi, NPL; 4 Department of Obstetrics and Gynaecology, Gorkha Public Hospital, Ghorahi, NPL

**Keywords:** vas score, constant-murley shoulder score, intra-articular steroid injection, manipulation under anesthesia, frozen shoulder

## Abstract

Introduction: Frozen shoulder, also known as adhesive capsulitis, is characterized by the insidious onset of pain and progressive loss of global active and passive mobility of the glenohumeral joint, which can be treated by non-surgical or surgical methods. This study was done to evaluate the functional outcome of intra-articular (I/A) steroid injection and manipulation of the shoulder joint manipulation under anesthesia (MUA) in frozen shoulder.

Methods: A cross-sectional study was done in a tertiary care hospital in Pokhara, Nepal. A total of 35 patients over 40 years were included in the study. All the patients underwent I/A steroid injection and MUA. The functional outcome was evaluated using a Constant-Murley shoulder score (CSS) and a visual analogue scale (VAS) scoring system.

Result: The mean age was 53.46±7.524 years. Twenty-two were female, while 13 were male. Twelve patients were associated with diabetes. Minor complications were noted in 15 patients (14 with transient pain and one with facial flushing). At 12 weeks, the mean VAS score was 2.16±1.33 cm, and the mean CSS score was 82.66±7.008. At 24 weeks, the mean VAS was 1.534±1.195 cm, and the mean CSS score was 85.77±6.998.

Conclusion: This study shows that patients with frozen shoulder treated with I/A steroid injection and MUA give excellent functional outcomes in most patients.

## Introduction

The shoulder is the most mobile and complex joint in the human body [1]. Frozen shoulder was coined by Codman in 1934 and is characterized by the insidious onset of pain and progressive loss of global active and passive mobility of the glenohumeral joint [2]. Later, Neviaser noted that the pathology of this condition was actually located in the capsule of the shoulder joint and therefore called it ‘adhesive capsulitis’ [3], commonly occurring at the age group of 40-70 years carrying greater risk for women (4:1). Diabetes and non-dominant arm are common and other associated factors are trauma, prolonged immobilization, thyroid disease, stroke, or autoimmune disease [4,5]. Diagnosis mainly depends on history and clinical examination, whereas other modalities of investigation such as X-rays, CT scans, and MRI scans are helpful.

Frozen shoulder is a frequent musculoskeletal ailment that affects up to 5.3% of the population, according to research, though the exact frequency and incidence rates remain unknown [6,7]. The disease lasts for an average of 30.1 months (ranging from 1 to 3.5 years), although it can last much longer, putting a substantial load on patients and healthcare professionals [8,9]. According to a recent narrative review, the thickening of the coracohumeral ligament (CHL), joint capsule, and synovium is a diagnostic marker for frozen shoulder [10]. However, no systematic review has yet combined data from imaging studies to pinpoint the intra-articular (I/A) and peri-articular changes connected to the disease [6].

It can be primary (idiopathic) and secondary with associated factors. The three clinical stages are the painful or freezing stage (three to nine months), secondly the frozen or stiffness stage (4-12 months), and lastly the thawing or recovery stage (12-24 months) [11]. Prevention is mainly by early mobilization. Although thought to be self-limiting, treatment includes nonsteroidal anti-inflammatory drugs (NSAIDs), I/A steroid injection, physical therapies, distension arthrography, manipulation under anesthesia (MUA), or arthroscopic release. In these patients, the main aim of treatment is pain minimization and restoration of shoulder mobility. Educating patients about the natural course and recovery of the disease motivates them to perform home exercises with muscle-relaxing adjuvants such as ice, heat, and electric or ultrasound stimulation to enhance the role of exercise. Operative treatments can be considered if non-surgical methods fail to achieve the desired treatment goal. All the patients in this study were given I/A corticosteroid injection mixed with local anesthetic, 2% plain lignocaine. The rationale of this mixture was to reduce inflammation and provide some analgesic effect after manipulation. As physiotherapy is the prime factor for its treatment, immediate pain relief is a major determinant for the patients to follow it. A practical approach of I/A injection of lignocaine and steroids followed by stretching exercises and joint mobilization is a better method because it is a quick and simple procedure [12].

Since frozen shoulder is common in individuals of the fifth decade and above, this is debilitating for the individuals. Our study is aimed at knowing the effectiveness of the treatment by MUA and I/A steroid injection. This cost-effective treatment can be a guide for the management of frozen shoulders in the future.

Evaluating the outcome of this treatment modality or any other treatment modalities can vary, and false-positive results can be obtained due to the varied natural history of the disease and self-limiting nature.

## Materials and methods

This cross-sectional study was conducted in a tertiary hospital for one year, and the sample size was 35 patients diagnosed with frozen shoulder. Ethical approval was received from the Institutional Review Committee, and written verbal consent was taken from the patients. All patients diagnosed with frozen shoulder and consenting to the study were enrolled.

Inclusion and exclusion criteria

Patients of age > 40 years and either sex with pain and stiffness of the shoulder for at least one month, restriction of active and passive range of movements (ROM) beyond 100° abduction and 50^° ^of external rotation as compared to the contralateral side, and no clinical improvement after one month of treatment with NSAIDs and physiotherapy were included. Patients were excluded if they had problems such as a history of cancer or rheumatoid arthritis, surgery or previous trauma on the shoulder, patients with severe neurological deficit of involved upper extremity, and improvement in ROM > 80% with NSAIDs and physiotherapy.

A standardized physical examination with ROM measurements was performed, and standard antero-posterior and lateral radiographs were obtained for all patients. The severity of frozen shoulder was assessed according to the Constant-Murley shoulder score (CSS). Preoperatively, the patients were treated with NSAIDs and physiotherapy for four weeks.

The patients were taken to the operation theatre after obtaining an informed written consent for the procedure. General anesthesia was administered, and the affected shoulder was painted and draped. Injection methylprednisolone 80 mg mixed with 5 cc of 2% lidocaine was injected in the affected shoulder joint as per posterior approach under full aseptic conditions, and the joint was manipulated gently in all directions. The sequence of manipulation was flexion, extension, abduction, adduction, and rotation (internal and external). An intramuscular injection of diclofenac sodium 75 mg was given, and the affected limb was immobilized in a collar-to-cuff sling for one to two days. In addition, NSAIDs and oral steroids were given for seven days. The post-manipulation protocol included progressive shoulder joint mobilization with a physiotherapist's help for half an hour daily for four weeks. The patients and their visitors were taught about the modality of physiotherapy and to continue it for four weeks. Patients were followed up at regular intervals of one month and were evaluated with the CSS and visual analogue scale (VAS) scores.

## Results

The mean age of the study sample was 53.46±7.524 years ranging from 41 years to 70 years. Out of 35 patients, 22 (62.9%) were females, and 13 (37.1%) were males. Most patients were right-handed. Twenty-five (71.4%) patients had involvement on the left side (non-dominant), and 10 (28.6%) had on the right side (dominant), which shows that involvement of non-dominant hand is more common. This study’s duration of symptoms ranged from five weeks to 14 weeks (mean: 8.17±2.63 weeks). Among 35 patients, 12 (34.3%) patients were known cases of type 2 DM and were under regular medications. Among the manipulated patients, the majority, 20 (57.2%), could return to work within 8-10 weeks. In this study majority, 20 patients did not have any complications after manipulation. Fourteen patients had complications in the form of transient pain, and one patient had complications in the form of facial flushing. No other serious complication was observed.

The CSS recorded before manipulation, after 12 weeks of manipulation, and after 24 weeks of manipulation, ranged from 45 to 75, with a mean score of 59.86±8.53 before manipulation. At 12 weeks, the mean score was 82.66±7.01 (p<0.05 at 95% CI), with a range of 65-94. At 24 weeks, the mean score was 85.77±6.99 (p<0.05 at 95% CI), with a range of 68-95. The score was higher at 24 weeks compared to that at 12 weeks (p<0.05 at 95% CI) (Table 1).

**Table 1:** Constant-Murley shoulder score (CSS) pre-manipulation, at 12 weeks, and at 24 weeks

CSS	Minimum	Maximum	Mean±SD	Significance (at 95% CI)
Pre-manipulation	45	75	59.86±8.527	
12 weeks	65	94	82.66±7.008	0.000
24 weeks	68	95	85.77±6.988	0.000

The VAS score recorded before manipulation, after 12 weeks of manipulation, and after 24 weeks of manipulation ranged from 4 cm to 8.5 cm, with a mean score of 6.13±1.25 cm before manipulation. At 12 weeks, the mean score was 2.16±1.33 cm (p<0.05 at 95% CI), with a range of 0.8 cm to 5.5 cm. At 24 weeks, the mean score was 1.53±1.19 cm (p<0.05 at 95% CI), with a range of 0.2-4.5 cm. The score was lower at 24 weeks compared to that at 12 weeks (p<0.05 at 95% CI) (Table 2).

**Table 2:** Visual analogue scale (VAS) pre-manipulation, at 12 weeks, and at 24 weeks

VAS score	Minimum (cm)	Maximum (cm)	Mean ± SD (cm)	Significance (at 95% CI)
Pre-manipulation	4.0	8.5	6.131±1.2249	
12 weeks	0.8	5.5	2.160±1.3311	0.000
24 weeks	0.2	4.5	1.534±1.1956	0.000

## Discussion

Codman described frozen shoulder as "difficult to define, difficult to treat, and difficult to explain" [13]. Frozen shoulder usually affects women in the sixth decade of life, frequently involves the non-dominant extremity, and occurs bilaterally in as much as 34% of patients. These epidemiological conclusions are well supported in the literature [14]. Although frozen shoulder is thought to be a benign self-limited disorder that resolves in one to two years, the authors suggest that patients with significant stiffness are good candidates for MUA rather than conventional treatment because conventional treatment of supervised physiotherapy programs needs to be carried out for months to years to regain range of motion [8]. Documented treatment options in the literature included benign neglect, supervised physical rehabilitation, NSAIDs, oral corticosteroid, I/A injection, distension arthrography, closed manipulation, open surgical release, and more recently arthroscopic capsular release [15]. Although there have been several treatment approaches for adhesive capsulitis mentioned in the literature, none of them has proven to be superior to the others [16]. Because it is most routinely employed by all surgeons, MUA has been considered the gold standard of treatment for this problem [17]. I/A injection can be done anteriorly, posteriorly, or laterally. Therefore, in patients who have had MUA, only a few weeks of supervised physiotherapy can help them acquire a functional range of motion [18].

In our study, most patients were elderly females, which is comparable with the study done by Amir-Us-Saqlainet al. [18], Farrell et al. [19], and Jacobs et al. [17]. From this, we can infer that frozen shoulder generally occurs in the elderly. The duration of symptoms is debatable according to different studies. In our study, it was 8.17±2.63 weeks ranging from five to 14 weeks. Complications were minor and insignificant in this study. In our study, the mean CSS was found to be 59.86±8.53 before manipulation, 82.66±7.008 (p<0.05) at 12 weeks, and 85.77±6.988 (p<0.05) at the end of 24 weeks. Oh et al. reported a mean CSS of 80 (p<0.05) at 12 weeks [20]. In the study by Wang et al., the average CSS was 70.1±6.2 (p<0.05) [21]. The study conducted by Dodenhoff et al. showed a mean constant score of 69 at 12 weeks and 73 at the end of 24 weeks [22].

In our study, the mean VAS score was found to be 6.131±1.22 cm before manipulation, 2.16±1.33 (p<0.05) at 12 weeks, and 1.53±1.19 cm (p<0.05) at the end of 24weeks. In a study by Oh et al., the mean VAS score was 1.8 cm at the end of 12 weeks (p<0.05) [20]. Similarly, Jacobs et al. in their study concluded that the mean VAS score was 1.15 (p<0.05) at the end of 16 weeks after MUA, which is comparable with our study [17]. However, in the study by Khan et al., the mean VAS score was 0.25 cm at the end of 12 weeks (p<0.05) [23]. Quraishi et al. observed that I/A steroid injections alone showed good results in terms of pain relief and mobilization in comparison to those receiving MUA [24]. 

None of our patients developed recurrence during the study period; however, it is difficult to comment on its recurrence due to the short duration of the study and follow-up. The design of the study was a hospital-based cross-sectional study, which limits its ability to determine any cause-effect relationship.

## Conclusions

The issues of frozen shoulder along with poor treatment can make it difficult for the treating doctor and the patient to achieve the desired outcome. Depending upon the expertise of the doctor, I/A steroid injection and MUA have the advantage over other mentioned methods as it is a minimally invasive procedure of short duration and minimal complications and act by releasing the thickened joint capsule as well as reducing the pain in the future and improvement in ROM of the shoulder joint. The functional outcome of I/A steroid injection and MUA is excellent and promising in most cases. Care has to be taken during MUA and I/A injection to minimize complications. Regular post-manipulation physiotherapy enhances the early return to function. Hence, I/A steroid injection and MUA are good choices and are advisable for the treatment of frozen shoulders.
